# Community Walks: a cluster randomized controlled trial of a multilevel physical activity intervention for low income public housing residents

**DOI:** 10.1186/s12889-023-16574-y

**Published:** 2023-08-31

**Authors:** Lisa M. Quintiliani, Julien Dedier, Marislena Amezquita, Melibea Sierra-Ruiz, Dariela Romero, Jennifer Murillo, Sarah Mahar, Melody Goodman, John B. Kane, Doreen Cummings, Timothy G. Woolley, Iolando Spinola, Scott E. Crouter

**Affiliations:** 1 Boston University, Chobanian and Avedisian School of Medicine, 801 Massachusetts Ave, Boston, MA 02118 USA; 2 Boston Medical Center, Section of General Internal Medicine, 801 Massachusetts Ave, Boston, MA 02118 USA; 3 School of Public Health, Boston University, 715 Albany St, Boston, MA 02118 USA; 4 Department of Biostatistics, School of Public Health, New York University, 708 Broadway, New York, NY 10003 USA; 5 Grants and Strategic Partnerships, Boston Housing Authority, 52 Chauncy St, Boston, MA 02111 USA; 6 Trinity Management Company, LLC, 75 Federal St. Floor 4, Boston, MA 02110 USA; 7 IEQ Technology, 115 W 8th Ave #370, Eugene, OR 97401 USA; 8 WalkMassachusetts, 50 Milk St. 16th Floor, Boston, MA 021109 USA; 9 Department of Kinesiology, Recreation, and Sport Studies, The University of Tennessee Knoxville, 1914 Andy Holt Avenue, Knoxville, TN 37996 USA

**Keywords:** Physical activity, Walking groups, Multilevel intervention, Cluster randomized trial, Public housing

## Abstract

**Background:**

Physical activity behavioral interventions to change individual-level drivers of activity, like motivation, attitudes, and self-efficacy, are often not sustained beyond the intervention period. Interventions at both environmental and individual levels might facilitate durable change. This community-based study seeks to test a multilevel, multicomponent intervention to increase moderate intensity physical activity among people with low incomes living in U.S. public housing developments, over a 2 year period.

**Methods:**

The study design is a prospective, cluster randomized controlled trial, with housing developments (*n=*12) as the units of randomization. In a four-group, factorial trial, we will compare an environmental intervention (E) alone (3 developments), an individual intervention (I) alone (3 developments), an environmental plus individual (E+I) intervention (3 developments), against an assessment only control group (3 developments). The environmental only intervention consists of community health workers leading walking groups and indoor activities, a walking advocacy program for residents, and provision of walking maps/signage. The individual only intervention consists of a 12-week automated telephone program to increase physical activity motivation and self-efficacy. All residents are invited to participate in the intervention activities being delivered at their development. The primary outcome is change in moderate intensity physical activity measured via an accelerometer-based device among an evaluation cohort (*n=*50 individuals at each of the 12 developments) from baseline to 24-month follow up. Mediation (e.g., neighborhood walkability, motivation) and moderation (e.g., neighborhood stress) of our interventions will be assessed. Lastly, we will interview key informants to assess factors from the Consolidated Framework for Implementation Research domains to inform future implementation.

**Discussion:**

We hypothesize participants living in developments in any of the three intervention groups (E only, I only, and E+I combined) will increase minutes of moderate intensity physical activity more than participants in control group developments. We expect delivery of an intervention package targeting environmental and social factors to become active, combined with the individual level intervention, will improve overall physical activity levels to recommended guidelines at the development level. If effective, this trial has the potential for implementation through other federal and state housing authorities.

**Trial registration:**

Clinical Trails.gov PRS *Protocol Registration and Results System*, NCT05147298. Registered 28 November 2021.

## Background

Half of all U.S. adults do not participate in regular physical activity and women consistently report less activity than men [1, 2]. Among women, Latinas and Black women report the lowest rates of meeting physical activity guidelines compared to white women (40.5% and 36.1% versus 49.6%, respectively) and are disproportionately affected by chronic conditions such as diabetes and obesity [1, 3]. Many studies have consistently demonstrated a significant causal link between physical activity and health-related outcomes under controlled- and free-living conditions [4–9].

Evidence suggests that characteristics of the environments in which people live can facilitate or inhibit physical activity [10, 11]. Low-income neighborhoods are more resource poor for the promotion of physical activity compared to higher income neighborhoods, exposing residents to environments that fail to promote physical activity [12, 13]. Inadequate physical activity opportunities, lack of proximal retail businesses, poor neighborhood aesthetics, high crime, and heavy traffic are all characteristics of low-income neighborhoods that have been shown to deter physical activity [14–16]. The negative effects of environmental factors on physical activity and obesity are amplified if low-income areas are in urban zones [12]. The physical environment has also been associated with healthy physical activity in several studies; [17–19] for example, access and safety encourage walking trail use [20]. Overall, research suggests that the physical environment is a powerful influence on behaviors [21–23].

On the other end of the spectrum, individual level interventions delivered one-on-one can also be efficacious in changing physical activity behaviors [24, 25]. eHealth programs have particular benefits including convenience, scalability, and ability to deliver tailored messages. Indeed, individual level interventions can incorporate a range of behavioral change techniques when promoting physical activity, with one systematic review reporting that information about health consequences, behavioral goal setting, self-monitoring of behavior, and encouraging practical social support were the techniques most commonly included [26].

A limitation of past research is the under-recognition that individual-level programs may be most effective when delivered in environments that support and maintain the promoted physical activity changes, thus prompting calls for intervention programs that combine individual- and environmental-level changes [27–31]. This combination is in line with mainstay public health frameworks including the socio-ecological model in which behavior is conceptualized as being influenced by multiple levels ranging from individual, inter-personal, community, and policy [32]. Only a few published evaluations of multilevel interventions exist that measure changes in physical activity, with some trials on-going [33–36].

We propose to evaluate the effects of a multilevel community-based intervention package to increase moderate intensity physical activity using a cluster randomized design, using public housing developments as the unit of randomization. We expect that delivery of an intervention package targeted to the environmental and individual levels will improve overall physical activity levels to recommended guidelines at the housing development level [31].

## Methods/design

### Participants

The study setting is family-designated public housing developments in the City of Boston managed by the Boston Housing Authority (*N=*16) and other housing developments serving low-income populations managed by a private management company (*N=*26). Boston Housing Authority developments provide housing for nearly 13,000 low-income people who identify as white (9%), Black (33%), Hispanic (51%), or other (7%). Approximately two-thirds of adult residents are women. About 90% of residents speak English and/or Spanish. Prevalence of obesity and related health conditions are higher among individuals living in Boston's public housing developments compared to other city residents [37]. Approximately 62% of Boston public housing residents report insufficient physical activity [37] and, qualitatively, report environmental structural barriers to physical activity [38].

### Design of the Community Walks study

The design of this study, called Community Walks, is a prospective, cluster randomized controlled trial with housing developments as the unit of randomization. A total of 12 developments (8 managed by Boston Housing Authority and 4 privately managed) will be recruited to participate. Four arms will be compared: environmental intervention alone (E) (*n=*3 developments), individual intervention alone (I) (*n=*3 developments), environmental plus individual intervention (E+I) (*n=*3 developments) and an assessment only control group (*n=*3 developments). All intervention activities assigned to a particular development are available to all residents of that development.

To evaluate our outcomes, a subset of participants from each development will be recruited to serve in an evaluation cohort. The cohort will be assessed at baseline, at one-year, and at two-year follow-up time points. Primary and secondary outcomes will be assessed at these times. Baseline data collection is anticipated to finish at the end of 2023, and final results are expected in 2025. Community Walks has received approval from the Boston Medical Center/Boston University Medical Campus Institutional Review Board. When research staff learn of an adverse event, they report the event to the PI who then assesses details of the event and relatedness to study procedures and fulfills reporting requirements as necessary.

### Formative research

From August 2020 to February 2021, we conducted four videoconference meetings with our established community advisory board comprised of Spanish- and English-speaking residents from Boston public housing developments. The first meeting was used to introduce the individual-level telephone physical activity program and ask members to elaborate on what they see as barriers to physical activity among public housing residents and to comment on barriers previously identified in the literature (e.g., lack of time, familial influences and responsibilities, lack of basic knowledge related to physical activity, lack of transportation, lack of resources and inadequate places to participate in physical activity, increased fatigue and lack of energy, and barriers related to body image). We also shared potential message content and asked for feedback on message acceptability and desired changes. In subsequent meetings, we provided revised content and received further feedback. Overall, members suggested simplifying information and sentence structure to aid understanding. They offered tips for making the telephone content more engaging, and suggested ways to recruit participants in their developments. We incorporated these suggestions into script development. Scripts were developed in English, translated into Spanish, and then reviewed and revised for cultural appropriateness by two bilingual team members. Meetings of the community advisory board continued to occur 2-3 times a year to provide updates and obtain feedback on study progress.

### Recruitment – public housing developments and residents

#### Recruiting public housing developments

Developments are eligible for selection as one of our study sites if they have at least 100 units. A statistician conducted the randomization stratified by management company (Boston Housing Authority/Private) using a computer program, such that 3 developments went to the E group, 3 developments went to the I group, 3 developments went to the E+I group, and 3 developments went to the control.

#### Recruitment and screening of the evaluation cohort

We propose to recruit 50 participants per development in the evaluation cohort. Eligibility criteria include being able to speak English or Spanish, being 18 years old or older, being a public housing resident from one of our study sites, not planning to move within the next 2 years, having access to a phone, not currently participating in another physical activity study, being able to walk independently without regular need for special equipment (wheelchair, scooter, or walker), ability to provide informed consent, and willingness to complete the accelerometer-based device measurement. By design, the entire cohort will be low-income.

Recruitment will take place at the developments, using common sampling rules and a direct household door-knocking approach [39–41]. Trained Research Assistants will approach units based on an assigned list and will attempt to identify an eligible resident using a brief script. The survey steps will be as follows: approach the unit, attempt to identify a resident, describe the study, determine eligibility, collect informed consent documents, collect the survey measurements, explain the physical activity device, and remind the residents of follow-up visits. Data collection documents for screening, documenting informed consent, and baseline, 12- and 24-month assessments are stored in REDCap [42].

### Intervention overview: role of the HLA

In all intervention developments, community health workers (termed Healthy Living Advocates or HLAs in this program) serve as intervention delivery agents and have been a successful way to partner with public housing communities for engagement activities and clinical trials [35, 43–45]. HLA training consists of 4 sessions (mix of in-person and Zoom) covering study overview and HLA responsibilities; information about importance of physical activity and influences on physical activity; data management and study protocol; culturally appropriate engagement; how to lead walking groups and adverse events. HLAs also participate in the hospital’s CPR/first aid program. At the conclusion of the training, HLAs undergo an evaluation and are provided with feedback on responding to safety-related situations, proficiency with engagement, and alternative exercises. Once proficiency has been reached, HLAs are able to lead activity sessions independently. HLAs are in frequent communication with each other and monthly with study investigators to discuss progress in each development and issues as they arise in the developments and with residents. At all meetings, HLAs will exchange ideas for engaging residents and strategize about problems that they are having in their work.

### Intervention overview

#### Environmental level intervention: improving the walkability of the public housing development environment

These activities aim to change multiple aspects of the housing development to enable walking as an easy, natural part of life. Our IRB determined that informed consent is not required of people who join environmental-level activities.

##### Walking trails and maps

WalkMassachusetts, a non-profit walkability and pedestrian safety advocacy organization serving the Commonwealth of Massachusetts, will mark walking trails surrounding each of the intervention developments with signage indicating walking trail status, duration, directions, and distance. There will be at least one short (15-20 min), one medium (25-30 min), and one longer (40-45 min) trail in each E and E+I development neighborhood, to accommodate walkers of various levels. HLAs will distribute maps of the walking trails and other highlights at every walk, at community events, and in the development’s management office. In addition to the walking trails, maps contain brief tips about physical activity and are available in both English and Spanish.

##### The process of developing the walking routes

Neighborhood walkability (obtained by assessment using the MAPS tool section 2.7.1) plus feedback from meetings with the managers, local community meetings, and the HLAs with lived experience in these neighborhoods, was used to inform the construction of the walking routes, and subsequent maps created by a professional graphic designer. These walking routes are designed to be used in the E and E+I intervention developments. With authorization from the City of Boston, wayfinding signage will be placed on poles and free-standing displays to instruct HLAs to accompany the walking routes illustrated on the maps.

##### Structured walking groups

HLAs will organize walking groups and adjust scheduling around work and childcare commitments of participants. Groups will begin meeting twice weekly and will occur more frequently as participation increases. The main recruitment strategies employed for these groups will be from flyers, from tables with posterboard displays, and by word-of-mouth referrals. Flyers will be distributed in a variety of ways including door-to-door, posting in high traffic areas, placing in mailboxes, and distributing at community events. Walk group participants will gather at a meeting place, such as a community room, within their development. Children are welcome to attend the walks with their parent/guardian to promote family participation and because improvised day care is not sustainable in the long run. Well behaved pets are also invited. All walking sessions will begin with light stretching. The groups then proceed with their walks. After each walk, they return to the meeting place for a post-walk stretching session. In case of inclement weather, all public housing developments have access to a large group room where they will lead indoor exercise classes, such as dance classes like Zumba or dances to upbeat popular music like Soca.

##### Advocacy training meetings

Leaders at WalkMassachusetts will host a 2-session advocacy training meeting at least once per year in each E and E+I development for the HLAs and interested residents. Residents will receive a $25 gift card for attending. The topics covered will be how to: evaluate the walkability of their neighborhood, report issues with sidewalks and other pedestrian infrastructure, and elevate issues to city officials, state agencies, and other advocate groups. Short-term results of this advocacy training could include cleaning up the development, restriping pavement markings, and installing pedestrian signage. Long-term changes might result from calling for city officials to improve sidewalks, adding trees, benches, incorporating complete street designs and traffic calming measures. Residents will know of and be able to connect with WalkMassachusetts for ongoing advocacy support after the training.

#### Individual level intervention: automated ehealth phone program for physical activity

Phone2Health is based upon a previously tested telephone program designed to increase physical activity among urban African American adults [46] and was further modified to meet the needs of public housing residents (see section 2.3). Phone2Health is a 12-week automated telephone-based program, consisting of sessions lasting 10 minutes or less, available in English and Spanish. Enrolled participants will receive one call per week for 12 weeks with a goal of increasing motivation and self-efficacy for physical activity. The calls will include conversations that speak to participants using computer-controlled speech. The calls are user friendly and interactive where participants can respond to conversations by pressing keys on their phone’s keypad. Each week various barriers and recommendations are addressed through several sections of the call. Each week the call starts with a brief physical activity assessment to assess the participants current level of physical activity. Participants' level of physical activity will be based on the previous week's goal and their ability to meet or exceed that goal. The call moves into a counseling section where participants are given feedback from their assessment, information and tips on physical activity, and the benefits of incorporating physical activity into their lifestyle. Topics addressed in this section may include injury prevention, identifying support systems, or discovering different types of physical activity. Following the counseling section is goal setting, where each week participants set a new and achievable goal such as adding an additional day of physical activity during the week. Changes to goals are monitored by the system and the program will suggest an adjustment if the participant has chosen to increase the goal by a large amount or to decrease their goal. The call ends with a call summary and upbeat concluding statement, such as an inspirational quote. The program utilizes a “call-out” mechanism in which registered users receive a weekly call at a predetermined time and day.

HLAs will facilitate access to the Phone2Health program for residents who live in developments randomized to receive the I and E+I intervention. The HLAs will obtain verbal informed consent from interested residents and set them up to use the telephone program by entering their phone number, preferred call days/times, and preferred language into a study-specific website. During enrollment, participants will indicate specific times and days they would like to be contacted. If a call is missed, the system will call back 15 minutes later. If that call is missed, the system will call on the second preferred day. After 2 weeks of missed calls, the HLA will contact the participant to troubleshoot any issues the participant is having (e.g., missed calls). Once completed, residents could re-enroll in the 12-week program if they wish to.

#### Combined intervention (environmental plus individual level)

The environmental plus individual intervention combines all activities of the environmental intervention and all activities of the Phone2Health program.

### Evaluation

Table 1 contains the assessment variables for Community Walks.

**Table 1:** Measures for the community walks study at baseline, 12-month, and 24-month follow up assessments

**Level**	**Variable**	**Description** *Objective=OB Self-report=SR*		**B**	**12 mo.**	**24 mo.**
	**Socio-Demographics**					
I	Personal characteristics & behaviors	Race/ethnicity, age, education, health literacy, smoking behavior	SR	X		
I	Food insecurity	*Household Food Security Survey Module Questionnaire* [47]	SR	X		
	**Outcome measures**					
I	Physical activity	*ActiGraph GT3X+ accelerometer-based device* (primary outcome) *Neighborhood Physical Activity Questionnaire* [48]: assesses time spent walking in and outside the neighborhood *Sedentary Behavior Questionnaire* [49]: assesses sedentary behaviors such was sitting while eating, reading, watching TV, etc.	OB SR SR	X X X	X X X	X X X
	**Mediators**					
I	Neighborhood Walkability	*Physical Activity Neighborhood Environment Scale* [50]: assesses public housing development related physical activity attributes, including presence of walking trails, interesting things to look at while walking, etc.	SR	X	X	X
I	Support from HLAs	*Modified Provider Support Measure* [51]: assesses four types of perceived social support: emotional/informational, readiness of change, identifying potential barriers, & positive social interaction	SR	X	X	X
I	Self-efficacy	*Self-efficacy physical activity scale* [52]: assesses confidence to participate in physical activity under various situations (*e.g.,* on weekends)	SR	X	X	X
I	Motivation	*Treatment Self-Regulation Questionnaire (TSRQ*) [53]: assesses motivation to do healthy behaviors and the degree of an individual’s motivation for a particular set of behaviors (*e.g.,* physical activity)	SR	X	X	X
I	Stages of change	*Stages of change for physical activity* [54]: assesses readiness to meet recommended guidelines for physical activity, categorized into five stages (pre contemplation, contemplation, preparation, action, maintenance)	SR	X	X	X
	**Moderators**					
E	PHD characteristics	Manager Interviews *Implementation Climate Scale for Evidence Based Practice* [55] Data from development verified through observation	SR SR OB	X X X		
E	Access to Physical Activity Resources	*Microscale Audit for Pedestrian Streetscapes-abbreviated tool *[56]	OB	X		
I	Neighborhood Social Cohesion	*Social Cohesion scale* [57]: assesses support from neighbors regarding perceived trust, willingness to help, degree to which values are shared, and connectedness among neighbors from residents’ perspective; related to physical and mental health factors among diverse populations [58, 59]	SR	X	X	X
I	Family	*Adaptation, Partnership, Growth, Affection, and Resolution (APGAR)* [60]: assesses level of satisfaction with family relationships & ability to confide in one another in times of trouble, communicate and share issues, support each other’s aspirations in life, and the way family expresses affection	SR	X		
I	Sleep	*Sleep Quality scale* [61]: assesses sleep quality and disturbance over a 7-day recall period	SR	X	X	X
I	Financial Strain	*Satisfaction with financial condition* [62] : assesses persons’ ability to meet payments on their monthly bills	SR	X		
I	Depressive Symptoms	*Center for Epidemiologic Studies Depression Scale (CESD) short form* [63]: assesses depressive symptoms	SR	X		
I	Quality of life	*EQ-5D-3L* [64]: assesses quality of life across domains mobility, self-care, pain/discomfort, and anxiety/depression	SR	X	X	X
I	Stress	*Perceived Stress Scale* [65]: 4-item measure	SR	X		
	**Process measures**					
I	Dose/Reach	HLA completed checklists, # completed Phone2Health calls	OB	X	X	X
I	Dose/Reach	Walking Group/Indoor Activity Form	OB	X	X	X

*I* Individual, *E* Environment

#### Environment-level assessments

We will conduct two types of assessments of the built environment at all study public housing developments. First, we will measure access to physical activity resources and walkability at all 12 public housing developments using the Microscale Audit for Pedestrian Streetscapes-abbreviated tool [56]. The second environment-level assessment is an assessment of public housing development characteristics conducted via an interview and survey of managers of the 12 enrolled public housing developments. The purpose of the interview is to identify development structure and processes that could affect the intervention. We will ask about the type of development (single building, apartments, high-rise, townhouses, etc.), the manager’s experiences working on health promotion activities, current or future physical activity initiatives, presence of any resident advocacy organizations, communication avenues with residents, and common walking areas for residents (to optimal map routes). At the end of the interview, we will administer the Implementation Climate Scale survey tool focusing on readiness to adopt evidence-based practice [55]. Using a structured form, adapted from existing tools [66, 67] study staff will also assess multiple characteristics of the development itself, including development size, number of buildings, structure of buildings, and other on-site facilities, such as courtyards and community gathering spaces. Together, this information was used in the creation of the walking routes and will serve as moderating variables (Table 1).

#### Individual-level measures: evaluation cohort

All eligible and consented participants will be sent a link to complete the surveys; if needed, the Research Assistant will administer surveys in person or over the phone. The Research Assistant will conduct the screening and baseline surveys and will also conduct the follow-up surveys at 12 months and 24 months for each enrolled participant. Standard socio-demographic measures will be assessed. All participants will receive a $40 pre-paid debit card at each completed assessment.

#### Primary outcome measurement: physical activity

Our primary outcome is minutes of moderate intensity daily physical activity. Participants will wear an ActiGraph GT3X+ accelerometer-based device on their hip and the device will be initialized to collect raw data at 30 Hz. The participant will be instructed to wear the device during waking hours every day for 7 days, except during water activities. At minimum, the GT3X+ must be worn for 10 waking hours/d on at least 4 days. Initially, the raw GT3X+ data will be used to estimate time spent in MVPA (physical activity at ≥ 3.0 metabolic equivalents units) using publicly available algorithms developed for adults [68]. The use of this algorithm will also allow for the detection of continuous walking and running bouts to help confirm participants engagement. All device data will be cleaned and processed [69] in R. As updated algorithms and machine learning models are developed, we will explore use of updated techniques to maximize the data outcomes.

#### Process measures

Process measures will be evaluated to monitor implementation of the intervention and to document receipt of the intervention. The HLAs will fill out a checklist after each physical activity event or promotion activity of the Phone2Health program. This information will help investigators determine intervention dosage and process. Specific metrics will include: 1) reach: the number of people who completed HLA walks/physical activity sessions or engaged in Phone2Health program; 2) the dose of the intervention: the number of HLA activities delivered and completed Phone2Health calls; and 3) participants’ feedback and perceptions of the intervention activities.

### Analysis plan

We will “deconstruct” the intervention for the measurement of adherence to the intervention protocol and use these adherence indices in secondary analyses. Adherence data will be collected based on 1) what, how much, and when individual elements were delivered in each development, and 2) what was reported as received by both study staff and HLAs and by residents. We will collect these data for all intervention levels and all components and will enter these data by level into regression models for intervention residents to determine the relative effects of different levels of intervention on physical activity behaviors. These variables will also be used to create cohorts for sensitivity analyses or stratified analyses.

Initial descriptive analyses will include an analysis of the baseline characteristics of participants. Data will be stratified with respect to study groups and the randomization assumption checked by comparing demographic characteristics across groups. Unbalanced characteristics will be included in multivariable models to control for any demographic difference between intervention groups.

#### Aim 1 (primary aim)

Hypothesis 1: Participants living in developments in any of the three intervention groups will increase minutes of moderate intensity physical activity more than participants in control group developments at 24 month follow up.

##### Within group analysis

Preliminary analysis will examine within group differences in each of the study groups from baseline to the end of the intervention at 2 years. Minutes of moderate or greater intensity activity per week will be examined using paired t-test if the distribution is approximately normal or the nonparametric Wilcoxon signed-rank test if normality cannot be assumed. McNemar’s chi-squared test will be used for a paired analysis of meeting recommended physical activity level (binary outcome) pre and post intervention.

##### Between group analysis

The between group hypothesis is tested in three separate bivariate analyses comparing each intervention group to the control group in two primary outcomes based on the cluster-randomized design. We examine change in each primary outcome pre-intervention to post-intervention: 1) the number of minutes of moderate or greater intensity physical activity per week (continuous); and 2) meeting the recommended guidelines of >150 minutes of moderate or greater intensity physical activity per week (dichotomous at individual level, proportion at group level). In addition, we will examine this hypothesis controlling for potential confounding factors and any variables that violate the randomization assumption using multilevel multivariable linear (continuous outcomes) and logistic (binary outcomes) regression models.

Hypothesis 2: Participants living in developments receiving the combined intervention (E+I) will demonstrate even greater increases in the number of minutes of moderate intensity physical activity per week (individual level) and the proportion meeting the recommended physical activity guidelines (development level) from baseline to 24-month follow-up, compared to either intervention delivered singly.

##### Statistical analysis

This hypothesis is tested in two separate bivariate analysis - one comparing the multilevel intervention group (E+I) to the environment intervention only group (E) and the other comparing the multilevel intervention group (E+I) to the individual level intervention only group (I).

Hypothesis 3: Participants living in developments receiving the combined intervention (E+I) will demonstrate even greater increases in the number of minutes of moderate intensity physical activity per week (individual level) and the proportion meeting the recommended physical activity guidelines (development level) from baseline to 24-month follow-up, compared to the sum of average change in environmental intervention compared to control and the individual level intervention compared to control (synergistic effect).

##### Statistical analysis

To examine this hypothesis, we exploit our study design which has a multifactorial design embedded in a cluster randomized controlled trial. Multifactorial experimental designs combine the rigor of experimental design with the ability to produce results on the effectiveness of alternate approaches to multicomponent interventions in a single experiment. We will use multilevel regression analysis and test for an interaction effect between the environmental and individual level interventions (E+I). The presence of a significant interaction between E and I on the primary outcome of physical activity suggests synergy between these components of the intervention. If the effects of the environmental level intervention and individual level intervention are additive there should not be a significant interaction in the model. In this case, we can compare the interaction results to the multilevel intervention group (E+I) to examine the exploratory hypothesis that E+I is more effective than E&I combined.

#### Aim 2: mediation/moderation

We will evaluate the mediator effects of motivation, self-efficacy, neighborhood walkability, support from HLAs, and fidelity and moderator effects (i.e., sleep, financial strain, public housing development characteristics, neighborhood social cohesion, and access to physical activity resources) on physical activity behavior.

##### Statistical analysis

Mediation and moderation are examined using variables at both levels, as such we will use multilevel structural equation models and path analysis to examine multilevel mediation and multilevel moderation within and across levels [70, 71]. The multilevel structural equation model framework is flexible and allows for testing models with predictors, mediators, and outcomes on either of the two levels. Given the small number of clusters both the Random Coefficient Prediction (RCP) method and the Latent Moderated Structural Equation (LMS) method with residual bootstrapping to estimate confidence intervals will be used to examine potential moderation. In RCP a random slope is predicted by a moderator. LMS can be applied to multilevel data by creating latent interactions among random coefficients.

##### Handling missing data

While the mechanism giving rise to the missing data cannot be determined by the observations, the sensitivity of parameter estimates to missing data assumptions can be studied by fitting multiple models that make different assumptions about the missing data process  [72]. For the missing data sensitivity analysis, we will compare three missing data approaches: 1) complete case analysis, 2) multiple imputation to estimate missing data values, and 3) mixed-effects pattern-mixture model that incorporates information about patterns of missing data, such as patterns of participant dropout. In the mixed-effects pattern-mixture model, participants are grouped according to the dropout patterns, and grouping indicators are included in the model similar to other person-level covariates. The model allows for the study of how responses vary by pattern and for generating an average profile that is weighted according to the missing data patterns.

### Sample size

#### Aim 1 - within group analysis sample size

A sample size of 21 achieves 90% power to detect a mean of paired differences of 3.0 with a significance level of 0.05 using a two-sided paired t-test. A sample size of 34 achieves >90% power to detect a difference of 3.0 with a significance level of 0.05 using a two-sided Wilcoxon Signed-Rank test. Assuming at least 20% of the sample changes between baseline and follow-up a sample size of 115 pairs achieves 80% power to detect an odds ratio of 3.0 using a two-sided McNemar’s test with a significance level of 0.05. The odds ratio is equivalent to a difference between two paired proportions of 0.10.

#### Aim 1 - between group analysis sample size

We power the multivariable analysis based on test for two means in a 2-level hierarchal design with level 2 randomization. Sample sizes of 96 subjects in each group, which were obtained by sampling 3 clusters with an average of 32 subjects per cluster, achieve >90% power to detect a difference between the group means of at least 5 minutes (or 5 percent difference in meeting recommended levels). A test based on a mixed-model analysis at a significance level of 0.05.

#### Aim 2 – mediation

The test of mediation effect is based on Sobel’s test. A sample size of 395 achieves 90% power to detect a mediation effect of at least 0.04 as measured by the product of two regression coefficients 0.20 (primary predictor) and 0.20 (mediator) when the significance level is 0.05.

#### Aim 2 – moderation

##### RCP method

A total sample size of 480 observations, were obtained by sampling 3 level-2 units (PHD) in the control group, 3 level-2 units (PHD) in I only group, 3 level-2 units (PHD) in the E only group, and 3 level-2 units (PHD) in the E+I group. Finally, 40 level-1 units (individuals with repeated measurements) were obtained from each level-2 unit (PHD). This sample achieves >90% power to detect a three-way interaction among the subject-specific slopes of at least 3.0. A test based on a mixed-model analysis assuming random slopes will be used. This test will be conducted at a significance level of 0.05.

##### LMS method

A total sample size of 492 level-1 units (individuals), which were obtained by sampling 3 level-2 units (PHD) in the control group, 3 level-2 units (PHD) in I only group, 3 level-2 units (PHD) in the E only group, and 3 level-2 units (PHD) in the E+I group, with an average of 41 level-1 units (individuals) per level-2 unit (PHD), achieve >85% power to detect an interaction difference among the group means of at least 3.75. A test based on a mixed-model regression analysis at a significance level of 0.05.

#### Aim 3 - qualitative interviews

We will conduct qualitative interviews with 40 key informants to explore the future implementation of the multilevel physical activity intervention using the Consolidated Framework for Implementation Research as our conceptual model [73]. We will purposefully sample key informants across multiple organizational levels to obtain the most relevant information. Sources will include HLAs, advisory board members, public housing development managers, and representatives from WalkMassachusetts, the Boston Housing Authority, the Boston City Health Department, and from Housing and Urban Development (the organization that administers public housing in the U.S.). Our guiding principle in sample size selection is purposeful sampling, which is used to gain information from information-rich cases intentionally selected to inform the research question.

Interviewers will follow a semi-structured interview guide with sections corresponding to each of the five CFIR domains: Outer Setting, Inner Setting, Intervention Characteristics, Characteristics of Individuals, and Process. The guide will allow for structured questions to be asked in all interviews, while allowing flexibility to probe or follow up on emerging topics. Interviews will be audiotaped, transcribed verbatim, and systematically read as they are being conducted to determine if information saturation has been reached. We will conduct a content analysis of the transcripts [74]. We will draw from the questions in our interview guide to create a preliminary coding framework. A staff member will independently code the transcripts according to the preliminary coding framework, adding additional codes as needed to cover emerging topics.

Interview themes will also be compared among those public housing developments in which key informants gave higher and lower organizational readiness ratings during the housing manager interviews conducted at baseline. Then, following team-based coding, the preliminary coding structure will be shown to the entire research team to review our analysis decisions. Codes will be added, refined, and deleted during this process. To enhance the trustworthiness of the analysis, we will hold at least two peer debriefing meetings with the entire research team to show them the transcripts and the codes applied and ask for their feedback [75]. Any discrepancies will be resolved through team discussion. Codes will then be summarized into themes. We will use NVIVO qualitative software to assist in data management.

## Discussion

The Community Walks study addresses a gap in the literature to test multilevel interventions in low-income populations and is nearly unique in its focus on this population, on a layered multilevel design, and on walking as the main form of physical activity. The environmental level intervention will be delivered by community residents trained as HLAs for leading walks using newly designed and marked walking trails around the development. Among residents randomized to the I and E+I condition, we will enroll interested residents to a more intensive individual level eHealth phone intervention to further promote motivation and self-efficacy for physical activity.

As described by Hall and colleagues, a continuum exists ranging from fundamental multilevel research to multilevel intervention research [29]. The Community Walks study represents an example of multilevel intervention research which “intervenes at two or more levels, measures factors at 2 or more levels, and examines interactions between levels” [29]. This type of research is designed to “inform decision making regarding levels, intervention components, and targets” [29]. Therefore, with our findings, we expect to contribute to a greater understanding of whether an individual or environmental intervention that includes an eHealth program and walking groups/trails and maps/and advocacy training are important drivers for physical activity promotion among urban public housing residents. This study will join other similar studies in contributing to this evidence base of multilevel physical activity trials, including the Steps for Change (on-going) – a trial testing a physical activity promotion program across personal, social, and physical environment domains among residents of senior public housing and developments [36]. The study will also add to the evidence base of community physical activity trials focused on walking groups, such as Positive Action for Today’s Health – a trial testing the efficacy of walking groups plus social marketing program [76].

The interviews we have planned at the conclusion of the trial will help us understand barriers and facilitators to intervention reach, adoption, implementation and maintenance, which will inform future intervention efforts. Other research teams have also included qualitative methods of intervention evaluation, including participatory evaluation (e.g., Ripple Effects Mapping) to elucidate factors most important to participants and other stakeholders [77]. Therefore, we expect the qualitative interviews to elucidate our trial findings and inform future directions of this work, which has implications for federal- and state-funded public housing developments nationwide. It is imperative to understand barriers and facilitators of uptake and adoption of health promotion programs, such as the Community Walks study in order to determine how to apply clinical trial findings to larger housing development networks, thereby achieving high public health impact [78].

Limitations of our study should be considered. Because our primary outcome is measured by actigraphy, those who are not able to ambulate independently are not included in the evaluation cohort. However, we do allow those using certain assistive devices (such as walkers) to participate in the evaluation cohort, as we thought actigraphy would be appropriate in these instances. In addition, only those willing to participate are included in the evaluation cohort. These may create bias in our sampling frame. Our program is available in English and Spanish, which represents most languages spoken in public housing developments. However, while people who speak other languages will be welcome to participate in E level intervention activities, they may not feel as welcome or be able to participate fully. Finally, the study is conducted in only one city, which may limit external generalizability.

In conclusion, a multilevel, multicomponent community-based study is being conducted to test whether individual- and environmental-level intervention programming promotes moderate intensity physical activity among urban public housing residents.

## Trial status

Protocol Version 1.8, June 7, 2023.

Recruitment Start Date: May 2, 2022.

Recruitment Approximate End Date: September 4, 2023.

## Data Availability

Lisa Quintiliani, Julien Dedier, and Melody Goodman have Access to the final trial dataset. No contractual agreements limit access to dataset. The datasets generated and/or analyzed during the current study are not publicly available due to the research is still ongoing, but are available from the corresponding author on reasonable request. Participant recruitment for this trial is not completed and is still ongoing.

## Abbreviations

E: Environmental
E + I: Environmental + Individual
I: Individual
HLA: Healthy Living Advocate
RCP: Random Coefficient Prediction
LMS: Latent Moderated Structural Equation
PHD: Public Housing Development
IRB: Institutional Review Board
TSRQ: Treatment Self-Regulation Questionnaire
APGAR: Adaptation, Partnership, Growth, Affection, and Resolution
CESD: Center for Epidemiologic Studies Depression Scale
